# Physicians’ role in the determination of fitness to drive in patients with Parkinson’s disease: systematic review of the assessment tools and a call for national guidelines

**DOI:** 10.1186/s40734-016-0043-x

**Published:** 2016-10-04

**Authors:** Onanong Jitkritsadakul, Roongroj Bhidayasiri

**Affiliations:** 1 Chulalongkorn Center of Excellence for Parkinson Disease & Related Disorders, Department of Medicine, Faculty of Medicine, Chulalongkorn University and King Chulalongkorn Memorial Hospital, Thai Red Cross Society, 1873 Rama 4 Road, Bangkok, 10330 Thailand; 2 Department of Rehabilitation Medicine, Juntendo University, Tokyo, Japan

**Keywords:** Parkinson’s disease, Driving, Fitness to drive, Driving competency

## Abstract

**Background:**

Physicians are usually at the forefront when the issue of driving ability is raised by Parkinson’s disease (PD) patients or their family members, even though few have been formally trained in this area.

**Objectives and methods:**

To identify relevant literature on driving assessment tools in patients with PD by performing a systematic review on this subject in order to provide background information for physicians on what types of driving assessment are available, and to delineate the role of physicians in providing fitness to drive recommendations.

**Results:**

Of 1,490 abstracts screened, 55 articles fulfilled the selection criteria that investigated assessment of driving ability in PD patients with questionnaires, off-road testing battery, driving simulators, and driving skill tests (on-road tests and naturalistic driving test). Despite different methodology across studies, PD patients were observed to commit more driving errors than controls. Poor driving performance correlated with motor, visual, and cognitive severity. Excessive daytime somnolence was common in PD drivers and the presence of falling asleep while driving was identified to be a significant predictor of car accidents.

**Conclusion:**

Although the evidence indicated more driving errors among PD drivers as identified by various assessment tools, the extent on how physicians should be involved in the evaluation process and make related recommendations remain unclear. Driving safety is an important public health issue in PD that needs better-defined specific legal and medical guidelines. National guidelines that establish risk assessment protocols involving multidisciplinary assessments are needed to assist physicians in making appropriate referrals for additional evaluations and recommendations when patients are deemed to be unsafe drivers.

**Electronic supplementary material:**

The online version of this article (doi:10.1186/s40734-016-0043-x) contains supplementary material, which is available to authorized users.

## Introduction

Driving a motor vehicle is a complex task that requires cognitive functions for decision-making and multi-level integration of sensory, motor, and cortical functions. Parkinson’s disease (PD) patients have impairment in both motor and cognitive ability that could lead to poor driving performance. Driving competence is, therefore, of great concern to PD patients, their families, healthcare professionals, and those with the responsibility of promoting and protecting public safety. As driving is an important activity of daily living for PD patients who rely on a vehicle for shopping and medical appointments, determination of driving competency in PD is essential and this determination requires a reliable assessment tool. Although the decision making process regarding fitness to drive is not entirely the responsibility of the physician, the issue of driving competency is often first raised by patients or family members during physician consultations. In response, physicians often feel uncomfortable providing recommendations due to a lack of established practice parameters or guidelines. Accordingly, this study set forth to conduct a review of the literature to identify the driving evaluations that are currently available and to provide practical recommendations to physicians regarding options and strategies they can discuss with PD patients when encountering the issue of determining fitness to drive.

## Review

The term “human mobility” is defined as a person being able to travel where and when he or she wants [1]. Fulfillment of mobility interests and desires produces physical, psychological, and social benefits, with private driving for many representing the ultimate in private mobility [1]. For those who drive, driving represents independence, self-reliance, freedom, and self-control. In contrast, loss of the ability to drive directly affects an individual’s mobility and this is associated with a number of negative consequences, including reduced outdoor activity, altered personal identity, decreased life satisfaction, increased depression, and increased dependence on family members or caregivers for assistance with transportation [2].

**Fig. 1:**
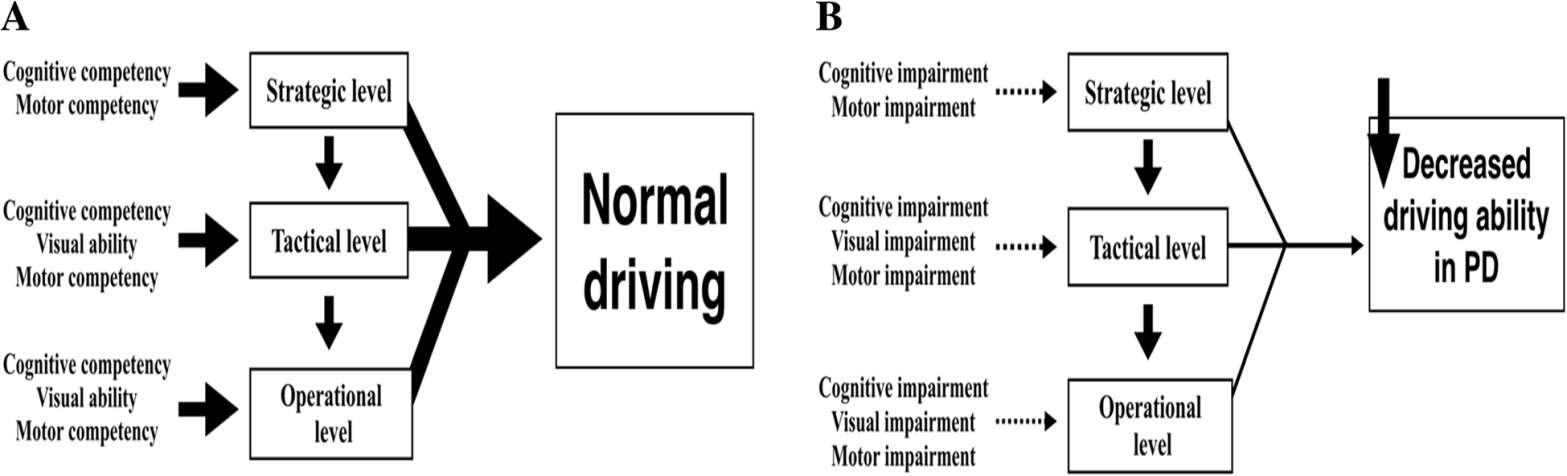
The model of driving behavior proposed by Michon (5). **a** The model of normal driving based on Michon’s driving behavior model. Drivers utilize three levels of skills in vehicle control: strategic (planning), tactical (maneuvering), and operational (control). Visual, cognitive, and motor skills are required for operations at tactical and operational levels of driving whereas visual skills are generally not required for operation at the strategic level of driving. **b** The model of driving behavior in Parkinson disease (PD) patients. PD compromises motor, visual, and cognitive performances in affected individuals. As a result, it is proposed that PD drivers have impairment of driving performance at all levels (operational, tactical and strategic levels). However, the severity varies with individual patients

Driving a car is a highly complicated task that is performed in a constantly changing environment and that involves integration of perception, information processing, attention, decision-making, motor programming, executive function, and concurrent task management [3, 4]. According to the driver behavior model proposed by Michon in 1985, drivers need to simultaneously conduct problem solving tasks that are divided into three levels of skill and control including: strategic (planning), tactical (maneuvering), and operational (control) levels (Fig. 1a) [5]. The strategic level is defined as general route planning, while the tactical and control levels involve individual responsiveness to driving circumstances with controlled or automatic action patterns respectively. Unsafe driving is defined as operating a motor vehicle in an unsafe manner, which often results in traffic-related violations [6].

### Effect of medical illness and Parkinson’s disease on driving ability

When determining fitness to drive in the elderly, the effect of aging needs to be considered as it can adversely affect driving competency with increased risk of unsafe driving, accidents, and injury [7–9]. According to the Global Status Report on Road Safety 2015, traffic accidents involving the elderly represent 1 out of 6 of the total of 1.2 million people who die worldwide each year in road traffic accidents [10]. Statistics from the US National Highway Traffic Safety Administration similarly reported that 16 % of all licensed drivers were 65 years and older who represented 17 % of all traffic fatalities [11]. These data illuminate the existential risks associated with driving among elderly drivers. However, the effect of aging is unlikely to be the sole reason for decreased driving competency among the elderly, given that neurological comorbidities, such as stroke, dementia, and PD, are common in this population. While existing data establishes the fact that patients with stroke and dementia are at significantly greater risk of road traffic accidents than age-matched controls, the evidence regarding the ability to drive among PD patients has started to accumulate and the findings show that they are also at substantially greater risk of unsafe driving [12–14]. Indeed, PD has recently been identified as one of the factors, together with old age and dementia, to be associated with driving restrictions in elderly men and women [7, 15]. Moreover, the presence of comorbidities in PD patients (e.g., heart and vascular diseases, eye problems, and other neurological disorders) can further compromise a patient’s driving ability, thus revealing another influential factor for physicians to consider when determining driving competency [16–18].

According to the driver’s behavior model, it is plausible that PD patients have impairment in all strategic, tactical, and operational levels (Fig. 1b). Motor deficits (e.g., bradykinesia, rigidity, tremor, and dyskinesia) may affect vehicle control (operational level) while non-motor deficits (e.g., cognitive decline, neuropsychiatric symptoms, and visual impairment) may impair route planning – both of which result in driving errors (strategic and tactical levels). This assertion is supported by numerous studies showing that PD drivers performed worse on testing of contrast sensitivity, cognition, sleepiness, and reaction time, when compared to controls [3, 19–23]. These poor performances in various domains are likely to influence their driving practices. For example, slowness in cognitive functions, including processing, choice reaction time, visual perception, and memory, have been identified as key factors that affect driving in PD patients [3]. One study indicated that up to 21 % of PD patients (32 out of 150 patients) stopped driving as soon as their diagnosis was made, mostly due to safety concerns (18.7 %). Interestingly, only 1.3 % (2 out of 150 patients) quit driving based on physicians’ recommendations [12]. In a large survey involving more than 5,000 patients with PD, one-third of patients reportedly restricted their driving in terms of reduced distance and trip duration due to their parkinsonian symptomatology, sleepiness, the effort required, and potential dangers [24]. They were also less likely to drive at night, in peak traffic, long distances, or alone [14]. As a result, about 18 % of PD patients who actively drove at baseline assessment ceased driving two years later, in comparison to only 3 % of control group drivers who stopped driving over the same period [25]. While earlier reports suggested that PD drivers had an increased risk of crashes per million miles traveled, one recent study yielded contradicting results indicating that PD drivers do not have a higher prospective risk of car crashes or incur a higher number of traffic violations compared to controls, which may be partly due to their restricted driving practices and a lower number of PD patients who continue to drive [12, 25]. Decreased driving ability and patient perception of their poor driving performance are likely to contribute to social isolation, sedentary lifestyle, and depression. As such, there is a need for physicians to identify patients who are at-risk and recommend them for regular driving assessment, driving retraining, or rehabilitation, if indicated and available. Identified from this systematic review, Table 1 provides a list of ‘red flags’ that will alert physicians on which patients should be evaluated for fitness to drive.

**Table 1:** The list of “red flags” that should alert the physician about PD patient’s fitness to drive

Methods	Red flags
1. Clinical history	• History of car accident (during the past 5 years)
	• Presence of sleep attacks while driving
	• High daily levodopa equivalent dosage ≥ 585 mg
	• Long disease duration ≥ 8 years
2. Questionnaire	• High Epworth sleepiness scale (ESS score ≥ 10)
3. Motor assessment	• High H&Y score ≥ 2.5 points
	• High UPDRS motor score > 27 points
	• High rapid pace walk score ≥ 6.22 points
	• High Webster’s scale
4. Cognitive assessment	• Low MMSE score < 27 points or low MOCA score
	• Poor performance on Trail A&B making test
	• Poor performance on complex figure test
	• Poor performance on block design test
	• Poor performance on dot cancellation test
5. Visual assessment	• High UFOV risk index ≥ 3
	• Poor performance on Pelli-Robson contrast sensitivity
	• Poor visual acuity

### Who determines fitness to drive in Parkinson’s disease patients?

Fitness to drive protocols vary greatly from country to country. While no uniform international standard exists, medical standards for driver licensing and physician’s guidelines for assessment of fitness to drive have been established in some countries, including the United States (American Medical Association and The National Highway Traffic Safety Administration), Canada (Canadian Medical Association), United Kingdom (Driver and Vehicle Licensing Agency), and Australia (National Transport Commission) [26–30]. Roles and responsibilities of drivers, health professionals, and driver licensing authorities have been proposed. While drivers have a responsibility to self-assess driving abilities and report any permanent injury or illness that may affect their ability to drive safely, health professionals have an ethical and legal duty to maintain patient confidentiality and must have legal justification for breaching that trust in cases where an unfit driver may pose a threat to public safety. Physicians that evaluate for fitness to drive and that identify a condition that may impair driving performance are obligated to report that finding to driver’s licensing authorities [26, 27]. The driver licensing authority is then tasked with the responsibility of deciding whether to license a driver or not, based upon the driver’s health report, driving performance record, and violation history.

Although risk determination may ultimately be the responsibility of licensing authorities, health professionals are generally responsible for recommending to licensing authorities whether individuals with medical conditions should be permitted to drive (with or without restrictions) [31]. Although guidelines have been published for health professionals regarding how to assess fitness to drive among drivers with underlying neurological disorders, most of those guidelines do not propose disease-specific tests, regulations, and severity of symptoms that would designate someone as an “unsafe driver”. This aspect is particularly important as patients with different diseases are likely to perform differently in their driving due to specific limitations caused by their underlying disorders. Although driving regulations have been established for epilepsy patients (UK: license is usually restored if there have been no seizures for 5 years and no other disqualifying condition, Australia: at least an annual evaluation for a conditional license for the epileptic patients who had no seizure for 12 months and stayed well with adherence to medications) and dementia patients (discontinuing driving if Clinical Dementia Rating scale is of 1 or above for patients with Alzheimer’s disease), no specific regulations have been implemented in patients with PD [6, 28, 32, 33]. The National Transport Commission of Australia recommends that physicians assess motor and cognitive functions of PD drivers, their response to antiparkinsonian medications, and monitor their symptoms every 12 months, but no specific course of action has been proposed for unsafe PD drivers [28]. Recently, a consensus statement was proposed for occupational therapy practitioners and driver rehabilitation specialists stating that PD patients with mild motor disability may be fit to drive with a recommendation for annual comprehensive driving evaluation while those with severe disease severity should discontinue driving [34].

### Driving evaluations in Parkinson’s disease patients: a physician’s role

In clinical practice, the first tier in the decision-making process of determining fitness to drive involves the treating physician (family physician or neurologist) when the issue of driving competency is raised by either family members or patients themselves on various occasions, for example when they are first diagnosed with PD or when they observe the effect of parkinsonian symptoms on their driving abilities [24]. At this stage, the physician is often asked to make an assessment regarding whether the patient is medically fit to drive, even though physicians are not trained in this area and current evaluation methods for determining driving ability in people with medical conditions or disabilities are very subjective, with information usually provided by patients, family/caregivers, and/or attending physicians [35]. While it is a physician’s legal responsibility in some countries or states to report unsafe PD drivers to the local authorities, this often becomes a point of conflict between physicians and patients, as evidenced by one survey indicating that while 77 % of physicians discussed driving with their patients, only 14 % of physicians reported patients to licensing authorities for further evaluation of driving competency [31]. It is also not surprising that one survey found a significant lack of knowledge among physicians regarding assessment for fitness to drive [36]. As one would expect, early-stage PD patients have less driving impairment than advanced-stage PD patients, but many early-stage drivers have difficulty accurately assessing their level of driving competency and some early-stage patients give up driving earlier than necessary [37]. Conversely, some advanced-stage PD patients who are at-risk to drive consider themselves fit to drive [3]. Classifying all individuals diagnosed with PD as an “unsafe driver” and preventing them from driving is not advisable due to the finding that a loss of driving privileges has been associated with psychological consequences that vary by patient [38, 39]. As such, we are faced with the challenge of finding a balance between what is best for the patient and what is best for the public. More specifically, we should become aware of tests and assessment protocols that accurately measure fitness to drive so that the best interest and safety of both PD patients and the general public can be protected.

In the medical literature, there are four main methods used for the evaluation of driving ability in PD patients, including questionnaires, off-road tests (clinical assessment and others), driving simulator, and driving skill tests (an on-road test and naturalistic driving) (Table 2). Different types of questionnaires were developed to assist physicians in their evaluation [12, 24, 40, 41]. However, there is poor correlation between patient questionnaires and disease severity scales in determining medical fitness to drive [12]. This discrepancy necessitates physicians to consider additional tests that evaluate driving-related skills and abilities, including vision, cognition, motor/somatosensory function, and neuropsychological testing (the so-called ‘off-road testing battery’) (Table 2) [6, 27, 28, 30]. While a combination of motor, visual, and cognitive assessments is often considered an adequate tool that determines functional ability of elderly drivers, it does not evaluate driving skills and does not predict the possibility of accidents in this population [26]. Due to a lack of standardized parameters and protocols for the different off-road testing methods, off-road tests alone cannot reliably predict actual driving performance or the likelihood that a PD patient is or will be at risk for a driving-related accident. Further, positive off-road testing results are not sufficient for designating someone an “unsafe driver” or recommending them for driving cessation [26]. Therefore, comprehensive driving evaluations often include on-road testing, which is considered to be the reference standard and the ultimate form of driving assessments (Table 2) [42, 43].

**Table 2:** Driving assessment tools in Parkinson’s disease

Types	Testing methods	Advantages	Disadvantages
Questionnaires and structure interviews	• Structured interview [53–55, 58] • The Epworth Sleepiness Scale [24, 40, 41, 56, 57, 59–61] • Restless legs syndrome questionnaire [61] • The sudden onset of sleep questionnaire [24] • SCOPA-sleep scale [62]	• Suitable for screening a large number of patients in a short period of time • Cost effective • Ability to capture subjective symptoms, e.g., sleepiness • No risk for physical injury during the test	• Lack specificity • Potential bias during recruitment. [12] • Findings may not be conclusive for final recommendations on driving.
Off-road testing battery	• Motor assessment (HY, UPDRS-motor, Webster’s scale, rapid pace walk, disease duration, LEDs, etc.) [4, 21, 25, 37, 75, 77, 82, 88, 91] • Cognitive assessment (MMSE, Trail making test, Complex figure test, Dot cancellation, block design test, etc.) [13, 67, 69, 74–76, 80, 83, 84, 86, 88, 91] • Visual assessment (UFOV) [13, 25, 75, 80, 82, 84, 91]	• The tests provide clinical information of patients on their ability in motor, cognitive and visual domains. • Some jurisdictions use an off-road evaluation to predict on-road behavior [27] • No risk for physical injury.	• Findings may not be conclusive for final recommendations on driving. • Findings are limited to clinical information on individual patients, not his/her driving performance.
Driving simulators	• Various types of driving simulators [4, 19, 20, 37, 63–74].	• Ability to control and standardize testing conditions and methods • Various outcome parameters can be implemented. • Patients are not exposed to significant risk associated with on-road tests.	• No standardized protocols • Simulator sickness • Testing scenarios are not real.
On-road tests	• An on-road test with/without instrument vehicle, and accompanied with a driver instructor for rating the driving score [3, 13, 14, 19, 21, 25, 74–91].	• Considered as a gold standard driving test for licensing new drivers by most authorities [43, 86] • Provided realistic driving test • Standardized outcome parameters	• Potential physical injuries and accidents during the tests • Unfamiliar testing scenarios • Not suitable for patients with physical limitations or handicaps
Naturalistic driving	• An attached devices equipped in a patients’ own car for collection of driving data [23, 92, 93].	• The most realistic driving test with familiar environment	• Potential physical injuries and accidents during the tests • Potential risk imposed to others on the road • No standardized testing protocols

Driving simulator determines driving abilities by providing driving stimuli and assessing driving responses in various challenging, but safe environments [44]. The inclusion of driving simulator as part of a comprehensive off-road test has been found to increase sensitivity and specificity of an off-road test in predicting pass/fail status of PD drivers on an on-road test [19]. Therefore, driving simulator may be an option for physicians to consider in patients who need driving evaluation but are deemed not to be suitable candidates for on-road testing for various reasons (Table 2). However, the testing protocols of driving simulators have not been standardized and their validity against actual road driving has not been established [44].

In an on-road test, patients drive on a real, predetermined road course for 45–60 min with a Certified Driving Rehabilitation Specialist (CDRS), advanced-specialized occupational therapist, or driver rehabilitation specialist that observes from the passenger seat [45, 46]. The observer is responsible for assessing patient driving performance, maintaining vehicle safety, and rating driving outcomes in a Global Rating Score (GRS) and a Sum of Maneuver Score (SMS). The GRS has 4 outcome grades, including pass, pass with recommendations, fail with potential for remediation, and fail. Although on-road tests are considered as the reference standard, there are certain drawbacks, including availability, an inadequate amount of safety assurance for at-risk patients, and potentially inadequate testing resources for PD patients that need to be re-tested and re-certified each year [42, 43]. The on-road test observer can provide both a comprehensive evaluation of driving skills and recommendations for car modifications or tools to keep someone driving safely for as long as possible [46, 47].

Naturalistic driving, also known as naturalistic observation, is a new method for evaluating driving skills and abilities. Naturalistic driving longitudinally monitors unsafe behaviors via instrumentation in naturalistic driving settings [48–51]. Naturalistic observation typically involves the use of the patient’s car, which is equipped with devices that continuously monitor various aspects of driving behavior, including information about vehicle movement, the driver, and the natural driving environment. This assessment method makes it possible to observe and analyze the interrelationship between the driver, and the vehicle, road, and other traffic in normal situations, conflict situations, and actual collisions [52].

### Systematic review of assessment tools for determining fitness to drive in Parkinson’s disease patients

The aim of this study was to perform a systematic review of the four main driving assessment methods (questionnaires and structured interview, off-road tests, driving simulator, and driving skill tests) and provide the evidence on individual methods for assessing driving ability in PD patients. To address the question of how much evidence we have regarding driving competency in PD, we performed a systematic review by searching MEDLINE, life science journals, Google scholar, and online books using the following key words: driving OR driving safety OR driving ability OR road test OR driving questionnaires OR sleepiness scale OR driving simulator OR naturalistic driving OR car sensor OR reaction time OR driver OR transportation OR automobile OR car OR vehicle OR collision injury OR car accident. Selected articles were required to have the term “Parkinson’s disease” AND any one of the above key words within the title and/or abstract.

A targeted search of the bibliographies of relevant articles was also performed to identify any additional studies for inclusion. Only original, full-text articles published in English between January 1973 and April 2016 that assessed methods for determining driving ability in PD were included in this review. Review and editorial articles were excluded. Two assessors (OJ, RB) independently screened each paper and agreement between the two reviewers were required for an article to be included in this review. A total of 1,490 titles and abstracts were reviewed, of which 124 full-length articles were selected for further review. Of those 124 articles, 59 articles fulfilled the selection criteria. Four of those 59 articles were then excluded as they were review articles. 55 full-length articles were finally included for critical evaluation (Additional file 1: Figure S1). A summary of studies involving driving in patients with PD are shown in Table 3. Findings were categorized according to the assessment tool used to determine fitness to drive in PD patients.

**Table 3:** Summary of studies involving driving assessment tools in patients with Parkinson’s disease

Types	Methods	Testing instruments	Main findings	Clinical recommendation
Questionnaires and structured interviews	Questionnaires delivered during interviews	Driving questionnaires [12, 53–55, 58, 62]	• PD drivers reported a high incident of collisions [12, 53, 62]. Patients with higher disease severity reported more collisions. • Up to 21 % of PD drivers gave up driving soon after diagnosis was made [12, 53] • Falling asleep while driving was a significant pre had found in PD drivers, and usually related with dopamine agonist medications [54, 55, 58]	• Appropriate as a screening instrument for physicians in routine clinical practice
		Epworth sleepiness scale [24, 40, 41, 56, 57, 59–61]	• Excessive daytime somnolence (EDS) and sleep attacks are more common in PD drivers than controls [40, 60, 61]. • EDS is associated with dopamine agonist medications [56] • PD drivers scored worse on ESS score than controls [60, 61] • Falling asleep while driving a car was a significant prognostic factor of car accidents [24, 59]	• ESS is a useful screening instrument for EDS and sleep attacks in PD patients. This test should be performed in PD drivers with history of daytime somnolence.
2. Off-road testing battery	Motor assessment	Hoehn & Yahr [3, 4, 13, 14, 19–21, 25, 37, 64, 70–75, 77–79, 81, 91]	• Greater HY score correlated with higher number of collisions or driving errors [37] • Greater HY score correlated with poor driving performance and a failed result with on-road tests [21, 25, 75, 77]	• HY scale should be part of the clinical evaluation in PD patients who come for fitness to drive assessment.
		UPRDS-motor [13, 14, 19, 20, 23, 25, 37, 64–67, 69–71, 73–80, 82, 91]	• High UPDRS-motor score correlated with greater of collision [37] • High UPDRS-motor score is a significant predictor of poor driving performance and fitness to drive [25, 75, 82]	• UPDRS-motor scale should be part of the clinical evaluation in PD patients who come for fitness to drive assessment.
		Rapid pace walk test (RPW) [75, 88]	• Poor rapid pace walk test correlated with poor driving performance [75, 88]	• RPW test may be considered as an off-road test in PD patients who come for fitness to drive evaluation. • More studies are needed to confirm its clinical validity.
		Webster’s scale [4, 63, 76]	• Poor Webster’s scale correlated with poor driving performance [4]	• Webster’s scale should be part of the clinical evaluation in PD patients who come for fitness to drive assessment.
		Disease duration and/or LEDs [13, 14, 19, 20, 23, 65–67, 69, 73, 79, 81, 82, 86, 87, 89, 91]	• Disease duration and/or LEDs did not correlate with driving performance.	• Disease duration and medication review should form part of basic clinical evaluation in PD patients at every visit.
	Cognitive assessment	MMSE [3, 13, 14, 20, 21, 25, 37, 65, 66, 69–73, 75, 77–79, 82, 85–88]	• Poor MMSE score correlated with higher number of collisions [37] • Poor MMSE scores correlated with poor driving performance. [86, 88]	• MMSE should be part of the clinical evaluation in PD patients who come for fitness to drive assessment.
		Trial A&B making test [13, 20, 21, 67–69, 72–75, 77, 78, 80, 82–84, 86, 87]	• Poor performance on Trail A&B making test correlated with poor driving performance and more driving errors [13, 67, 69, 75, 80, 83, 86]	• Neurocognitive tests should be considered in PD patients with cognitive complaints who come for fitness to drive assessment.
		Complex figure test [13, 20, 25, 83, 84]	• Poor performance on complex figure test correlated with poor driving performance. [13, 83, 84]	• Neurocognitive tests should be considered in PD patients with cognitive complaints who come for fitness to drive assessment.
		Block design test [68, 83, 86] [84],	• Poor performance on block design tests correlated with poor driving performance [84, 86]	• Neurocognitive tests should be considered in PD patients with cognitive complaints who come for fitness to drive assessment.
		Dot cancellation test [74, 76, 78]	• Poor performance on Dot cancellation test correlated with decreased driving ability [74, 76]	• Neurocognitive tests should be considered in PD patients with cognitive complaints who come for fitness to drive assessment.
	Visual assessment	UFOV [13, 19, 25, 68, 73–75, 78, 79, 82, 84, 87, 89, 91]	• Decreased UFOV score correlated with poor driving performance and higher collision risk [13, 25, 75, 80, 82, 84]	• Visual assessment with UFOV may be considered in PD patients who come for fitness to drive assessment.
		Pelli-Robson contrast sensitivity [23, 78, 79, 89, 91]	• Low-contrast visibility conditions imposed significant hazard for PD drivers.	• More studies are needed to confirm the validity of this test.
		Visual acuity [19, 20, 74, 75, 78, 91]	• Poor visual acuity limits driving ability in PD patients.	• Visual acuity should be performed in PD patients who come for fitness to drive assessment.
Driving simulators	Driving simulators (16 papers)	Driving simulators [4, 19, 20, 37, 63–74]	• PD drivers committed more driving errors than controls [4, 37, 63], • Greater PD disease severity determined with UPDRS or HY scale are correlated with poor driving performance [4] • Poor performance on cognitive test especially with executive testing and visual attention correlated with more driving errors [20, 37] • Driver assistance improved the driving performance in PD patients [69, 72]	• Physicians should consult local authorities on PD patients who may be unfit to drive for further evaluation.
4. Driving skill test	On-road tests (24 papers)	On-road tests [3, 13, 14, 19, 21, 25, 74–91]	• PD drivers performed worse on on-road tests when compared to controls [3] • Greater PD disease severity determined with UPDRS or H&Y scale correlated with poor driving performance [3, 82, 89] • Poor performance on cognitive and/or visual tests affect driving ability in PD patients [25, 82]	• Physicians should consult local authorities on PD patients who may be unfit to drive for further evaluation.
	Naturalistic driving (3 papers)	Naturalistic driving [23, 92, 93]	• PD drivers committed more errors, as shown by slow brake response time and slow reaction time [23]	• Physicians should consult local authorities on PD patients who may be unfit to drive for further evaluation.

1. Questionnaires and structured interview
  Driving assessment by questionnaires is the most practical method for evaluating fitness to drive if we aim for a large number of patients in a short period of time. While this method is potentially cost effective, it lacks specific information from individual patients based on the types of questionnaire. Of 55 articles, 14 studies assessed driving ability in PD patients using various forms of questionnaires being part of structured interviews [12, 24, 40, 41, 53–62]. Most questionnaires were composed of specific items that assessed driving performance, driving risk, and history of car accidents in addition to parkinsonian symptomatology. Information on daytime sleepiness and sleep attacks were included as additional items or inquired from the assessment with Epworth Sleepiness Scale (ESS), or SCOPA sleep scales. The main findings of these studies were consistent in reporting higher risk of car accidents among PD drivers than normal subjects. Importantly, most PD drivers also reported more daytime somnolence and sleep attacks than their matched controls when evaluated by sleep scales. The presence of falling asleep while driving was also found to be a significant predictor of car accidents [24]. While the risk of sleep attacks was highest with dopamine agonists, they were reported to be less with levodopa monotherapy.
  Although results of these studies supported significant driving risk among PD patients, these findings should not be generalized to imply that all PD drivers are unsafe. Careful interpretation of specific scenarios or additional assessment is generally recommended before final recommendation is made on individual patient.
2. Off-road testing battery
  A number of clinical tools were implemented as part of the off-road testing battery to assess driving-related skills in PD on motor, cognitive, and visual functions (Table 2). In all studies involving off-road tests, they were conducted as part of the evaluation panel together with one of the other assessment methods, including driving simulators, on-road tests, and naturalistic driving test. Significant correlations were observed between a set of motor and cognitive assessment and a number of collisions and poor driving performance as detailed in Table 3. The recommendations of these studies include Hoehn and Yahr (HY) staging and section III of Unified Parkinson’s Disease Rating Scale (UPDRS) for the assessment of motor function, the Useful Field of View (UFoV) for the evaluation of vision, and Mini-Mental Status Examination (MMSE), Trail A&B Making test, complex figure test, and block design test for cognitive assessment (Table 3). Similar recommendations were observed in a recent evidence-based review on driving in PD that include a subset of cognitive and visual assessments (e.g., UFoV, complex figure test) as probably predictive of driving performance [22].
  The impairment of motor, visual, and cognitive functions in PD patients as identified by these off-road tests was associated with poor driving performance, and higher number of collisions (Table 3). The utility of these off-road tests probably represents a set of screening tools that is available for physicians to perform during the initial assessment so it can provide them with objective information on motor, visual, and cognitive performances of individual patients before undergoing more detailed driving test. More studies are needed to determine whether off-road tests are comparable to on-road tests in identifying unsafe PD drivers. At present, in a practical sense, most of the off-road tests (e.g., motor and cognitive assessments) can be utilized by physicians on the spot in the outpatient setting when suspecting any PD patients to be unsafe drivers. In addition, they are widely available in most practices and medical centers, representing the first-tier of test battery for a determination of driving competency. Whether off-road tests are adequate on their own in identifying most if not all unsafe drivers (without a need to be confirmed by additional on-road tests) remain to be confirmed in future studies. Future studies are needed to determine cutpoints of risk factors with off-road performance (e.g., sensitivity, specificity, negative predictive value, and positive predictive value) and prospective crash risk. However, some of these off-road tests have been implemented in certain guidelines. For example, the recent consensus statements have adopted several findings of the off-road tests towards a recommendation of cessation of driving or additional evaluation by driving authorities [34].
3. Driving simulator
  Out of 55 articles, 16 articles assessed driving ability in PD patients using a driving simulator [4, 19, 20, 37, 63–74] (Table 3). Among these articles, two studies combined a driving simulator and an on-road test on the same target population [19, 74]. When tested with simulators under different driving conditions (high-contrast and low-contrast visibility conditions), PD patients committed more driving errors than controls, including delayed reaction time, steering accuracy, impaired vehicle control, red light violations, and higher number of collisions, [4, 63, 65–68, 71]. Moreover and as compared to controls, PD patients approached signals with a slower speed, drove more slowly around curves, and had more difficulty maintaining lane position around curves [66]. Poor driving performance in simulator was found to correlate with disease severity and executive functions, as determined by Webster, UPDRS, and HY scales [4, 37, 68–71]. Importantly, performance deficits in driving simulators were identified even in patients with mild-moderate severity when they were challenged under dual-task conditions [65, 66, 70].
  In general, driving simulator is a screening test option for at-risk driver that yields data and findings that traditional evaluation techniques cannot produce. In addition, simulators can also be used to determine predictors of driving performance that cannot be tested on the road due to ethical, safety, and practical concerns (e.g., night, high volume traffic, poor weather conditions) [22]. Predictors identified on the simulator can be further tested in the on-road tests [22]. In cases with poor performance from driving simulator, the physician should advise the patients to undergo an on-road test organized by a driver licensing authority.
4. Driving skill tests
  4.1) On-road tests
    An on-road test is capable of identifying tactical errors made when patients maneuver the vehicle in response to demands of the changing environment, in addition to strategic and operational errors made while driving. PD drivers demonstrated significantly higher on-road test failure rate and significantly more on-road driving errors than controls [75]. Out 55 articles, 24 articles assessed driving ability by on-road tests [3, 13, 14, 19, 21, 25, 74–91]. In addition to deficits identified by driving simulators, PD drivers had significantly more errors than controls for observing blind spots, backing up, parking, and negotiating traffic lights [14]. Other problems identified among PD drivers included use of mirrors and delays in decision-making and judgments [81]. Similar to findings from driving simulator-based assessments, PD drivers demonstrated poorer performance in the concurrent task of detecting roadside targets than controls [91]. What is truly considered poor driving performance can be difficult to interpret in these studies since not all studies provided pass/fail outcomes on the on-road tests [22]. One study that utilized CARA assessment as the main outcomes of pass (fit to drive without restriction) and fail (fit to drive with restrictions and unfit to drive) in 80 PD patients identified approximately one out of four patients failed the on-road test [19].
    Following a brief report showing that PD drivers with EDS may exhibit driving errors in driving simulators, five out of 21 PD drivers with self-reported EDS on ESS underwent an on-road test [64, 79]. Although neither EDS nor antiparkinsonian medications were associated with poor on-road driving performance, this study merits further investigation involving a larger number of patients with a more stringent methodology.
  4.2) Naturalistic driving test
    From our systematic review, three studies used the naturalistic driving method to examine whether PD symptoms (motor, cognitive, vision, sleepiness, depression) were associated with driving performance [23, 92, 93]. Naturalistic driving vehicles are equipped with several small cameras and sensors that continuously and inconspicuously register vehicle maneuvers, driver behavior, and external conditions. Compared to controls, PD drivers exhibited increased driving risks, as shown by slower brake response time, and slower reaction time, as well as having significantly more cognitive and depressive symptoms. In addition, PD drivers who had a poor perception of their health were more likely to restrict their driving due to worsening PD symptoms and more likely to have more noticeable declines in multiple driving-related abilities. Naturalistic driving provides insight into everyday driver behavior. This method facilitates observation and analysis of interrelationships between the driver and the vehicle, road, and other traffic in normal situations, conflict situations, and crashes.

## Conclusion

Due to the progressive nature of PD, as well as the heterogeneity of symptoms that fluctuate on a daily basis, adequate monitoring for fitness to drive is of paramount importance to ensure the safety of both PD patients and others on the road. A fitness to drive evaluation is needed to discern individuals with PD who can continue to drive safely from those who are likely to endanger themselves and others. However, the extent that physicians should be involved in the evaluation process, and make related recommendations remains unclear. Our systematic review indicates that not all the tools are available in routine clinical practice. When considered only questionnaires, structured interviews and off-road testing battery, most of the tests are part of standard clinical examination (e.g., HY, MMSE, UPDRS-motor) and can easily be tested on PD patients in clinical practice. The information obtained from these tests (e.g., cognitive status, severity of motor symptoms) provides important background information to local authorities when patients are proceeded to undergo additional tests, for example on-road tests. The situation is more challenging with driving simulators, on-road tests, and naturalistic driving because there are differences between studies involving these tools in technical characteristics, primary outcomes, and a lack of validity. These concerns probably limit the implementation of a uniform guideline on how these tests could be utilized as part of a formal assessment of driving competency in PD patients. Moreover, physicians may not be aware of the details of these tests and find it difficult to recommend them to PD patients. While on-road tests are considered by some as the reference standard, these tests are not as yet implemented or recommended by recent consensus statement and evidence-based review [22, 34, 43]. However, on-road tests are usually part of the formal assessment imposed by driving authorities. In certain situations, physicians may be able to refer patients directly for an on-road test or naturalistic driving if they are locally available and patients are deemed to be fit to undergo such tests by local authorities. Direct referral to an on-road test may be particularly useful when there are conflicting opinions between the patient and family members about their safety. We recommend that physicians consult their driving authorities for advice on additional tests if concerns are observed from structured interview and off-road tests.

**Fig. 2:**
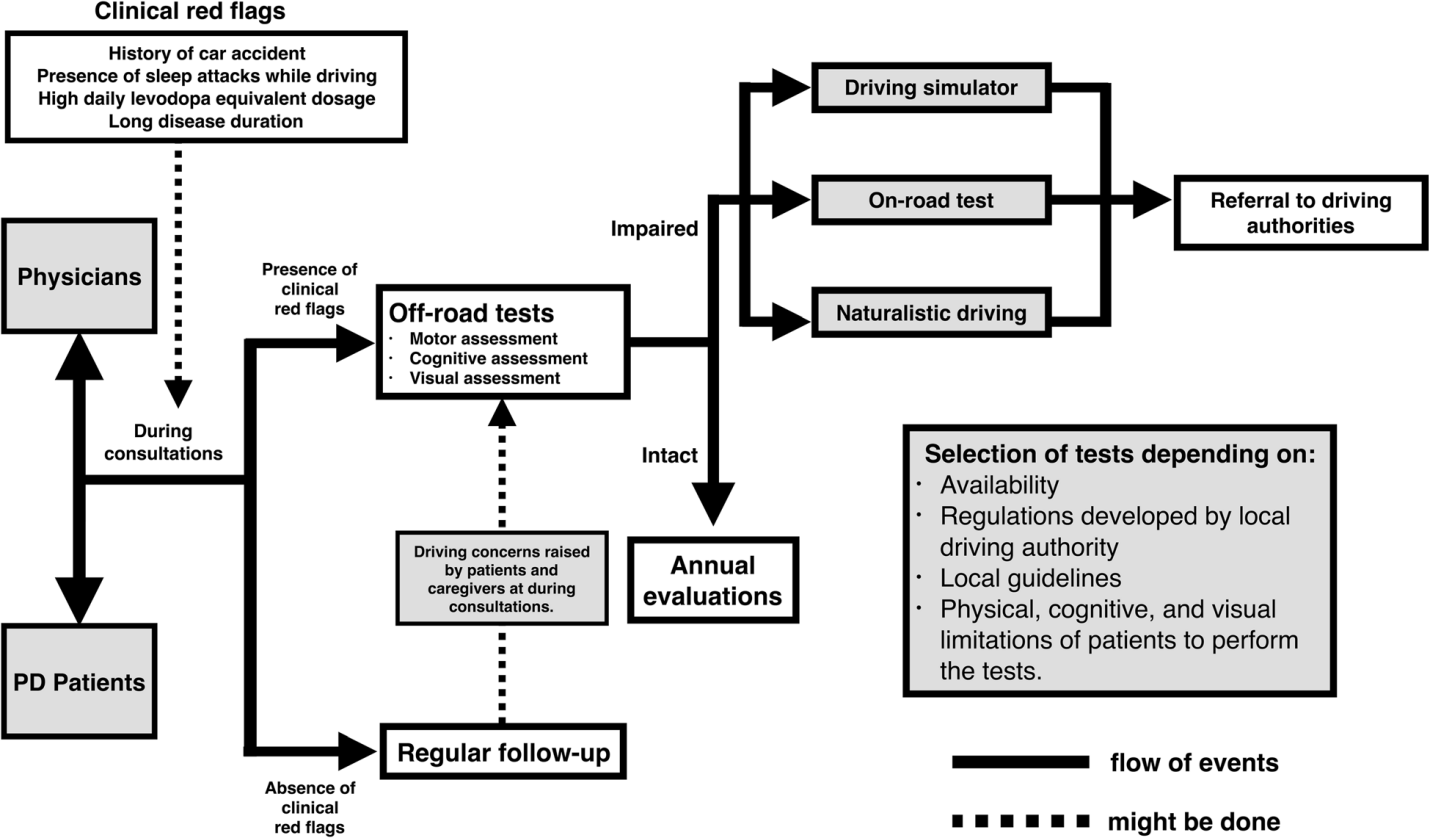
The diagram illustrating the role of physician in the determination of fitness to drive in patients with Parkinson’s disease (PD). In PD drivers who are deemed to be fit to drive, they can continue driving with an unconditional license. In cases where PD drivers were identified from ‘clinical red flags’ to be unsafe, they should undergo an off-road test which is composed of three major components to determine an individually driving ability, including motor, cognitive, and visual assessments. Some of these assessments (e.g., HY, MMSE) are part of standard clinical examination which can be performed by treating physicians during routine consultations. However, others (e.g., UFoV test, Pelli-Robson contrast sensitivity) may not be available locally and require additional referral. For PD drivers who passes an off-road test, they can continue driving as usual, but with a recommendation of annual evaluation. For PD driver who fails an off-road test, a physician might request for further evaluation for fitness to drive by using an on-road test, driving simulator, or naturalistic driving depending on patient’s conditions, availability, local guidelines, and regulations. HY: Hoehn & Yahr; MMSE: Mini-Mental Status Examination; UFoV: Useful Field of View

Based on experiences described in related reports, it appears that physicians are at the forefront when the issue of driving ability is raised by patients or their family members (Fig. 2). It is, therefore, necessary for physicians to have and conduct a risk assessment protocol that can evaluate if his/her patients are able to safely control and maneuver a motor vehicle. In addition to clinical assessments specific to PD, physicians should have sufficient knowledge about the functional capacity of the driver that is based on the minimum functional requirement for safe driving. In clear-cut or advanced-stage PD patients, assessment is not a problem since driving cessation is normally agreeable by all parties. Alternative modes of transportation should be explored for these patients. The problems tend to arise in those PD patients that are not clear-cut or advanced-stage in which careful evaluation and informed judgment on the part of treating physician is required. As a result, physicians are likely to make their judgment based on physical assessment rather than driving assessments, as they do not know how to undertake or participate in this kind of assessment. Given that most physicians lack basic knowledge and the formal training necessary to make a recommendation regarding which PD patients can safely operate a motor vehicle, we propose that this training be incorporated into the medical curriculum, as well as into continuing professional development programs [36]. The focus of the training should be on the following aspects: 1) the impact of PD for safe driving ability, 2) the basic knowledge on the legal requirement for PD patients on fitness to drive, 3) ability to treat, manage and monitor the individual’s PD condition with ongoing consideration of their fitness to drive, and 4) when to inform driving authorities when fitness to drive requires notification but an individual cannot or will not notify the local authority. At the same time, there is a need for more research to develop validated tools that can be implemented by licensing authorities as objective measures to determine fitness to drive that are specific to PD. Driving safety is an important public health issue that needs better-defined legal and medical guidelines, both generally and specific to PD drivers. The release of recent consensus statements on driving with PD in Canada is a promising development that should be expanded to other regions [34]. In addition, local guidelines should be communicated to physicians regarding assessment and reporting of PD drivers to the appropriate local agency when patients are deemed potentially unsafe. Once a patient is reported as unsafe, a well-defined course of action should be undertaken, including formal retesting for driver recertification by licensing authorities that used validated objective assessment tools (Fig. 2). The establishment and implementation of consistent and specific guidelines would help alleviate the conflicts experienced by physicians regarding patient confidentiality and help promote and improve public safety. PD drivers who are borderline in their driving competency should be offered driving rehabilitation or retraining that focuses on operational and tactical skills, as well as visual, cognitive, and motor functions so they can be reassessed for licensing if their skills improve [94].

## Additional file

Additional file 1: Summary of the search results. (JPG 1265 kb)

## Abbreviations

CDRS: Certified driving rehabilitation specialist
ESS: Epworth sleepiness scale
GRS: Global rating score
HY: Hoehn and Yahr Stage
MMSE: Mini-mental status examination
PD: Parkinson’s disease
SCOPA: Scale for outcomes of Parkinson’s disease
SMS: Sum of maneuver score
UPDRS: The unified Parkinson’s disease rating scale
