# Supplementary figures and images for: Physicians’ role in the determination of fitness to drive in patients with Parkinson’s disease: systematic review of the assessment tools and a call for national guidelines
Source: J Clin Mov Disord. 2016 Oct 4;3:14. doi: 10.1186/s40734-016-0043-x (PMC5048693; doi:10.1186/s40734-016-0043-x)

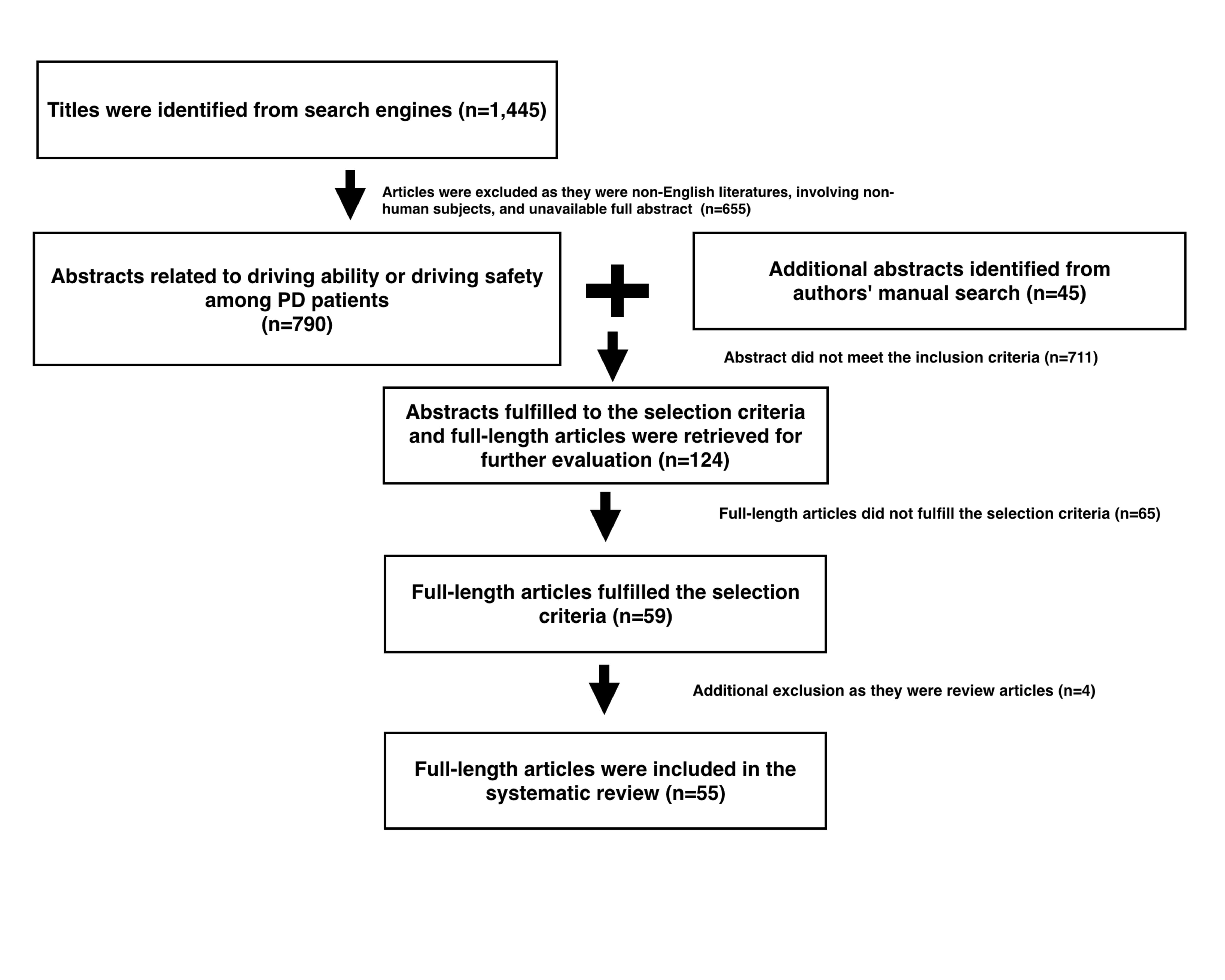

Supplement: Additional file 1: — Summary of the search results. (JPG 1265 kb) [file 40734_2016_43_MOESM1_ESM.jpg]
